# The Resistant Soybean-*Aphis glycines* Interaction: Current Knowledge and Prospects

**DOI:** 10.3389/fpls.2020.01223

**Published:** 2020-08-11

**Authors:** Martha I. Natukunda, Gustavo C. MacIntosh

**Affiliations:** ^1^ MacIntosh Laboratory, Roy J. Carver Department of Biochemistry, Biophysics and Molecular Biology, Iowa State University, Ames, IA, United States; ^2^ Department of Agronomy, Iowa State University, Ames, IA, United States

**Keywords:** soybean, soybean aphids, biotypes, host plant resistance, *Rag* genes, gene pyramiding

## Abstract

Soybean aphids (*Aphis glycines* Matsumura) are invasive insect pests of soybean, and they cause significant yield losses. Resistance to soybean aphids is conferred by Resistance to *Aphis glycines* (*Rag*) genes. Since the first discovery of aphid-resistant soybean genotypes in 2004, several studies have attempted to characterize *Rag* genes from aphid-resistant soybean genotypes. To date, 12 *Rag* genes and four quantitative trait loci for aphid resistance have been reported on soybean chromosomes 07, 08, 13, 16, and 17. Although candidate genes have been proposed for several discovered *Rag* loci, additional studies are needed to pinpoint, validate, and further explain the potential mechanisms of *Rag* gene action. A major challenge to utilizing host plant resistance is the discovery of virulent aphid biotypes that can colonize aphid-resistant soybean. This occurrence suggests the need for additional studies to devise strategies to enhance the effectiveness of aphid-resistant soybean. In this mini review, we discuss current knowledge on the resistant soybean-*Aphis glycines* interaction, potential mechanisms of *Rag* gene action, opportunities to discover new *Rag* genes, and prospects for utilization of host plant resistance to manage soybean aphids. A clearer understanding of host plant resistance to soybean aphids will guide researchers on strategies for developing soybean varieties with more durable aphid resistance, reducing the present challenge of virulent aphid biotypes.

## Introduction

Soybean aphids (*Aphis glycines* Matsumura) are important insect pests that can cause yield loss of up to 50% in soybean (*Glycine max* [L.] Merr.) by sucking plant assimilates using their piercing and sucking mouthparts (stylets) (Ragsdale et al., 2007; Beckendorf et al., 2008; Ragsdale et al., 2011; Tilmon et al., 2011; Bhusal et al., 2013). Cultivation of aphid-resistant soybean varieties is a preferred strategy for controlling soybean aphids (Ragsdale et al., 2011; Hodgson et al., 2012). Resistance to soybean aphids is conferred by Resistance to *Aphis glycines* (*Rag*) genes (Hill et al., 2004). To date, 12 *Rag* genes have been reported on chromosomes Gm07(*Rag1*, *rag1b*, and *rag1c*)(Li et al., 2007; Zhang et al., 2009; Kim et al., 2010b; Nurden et al., 2010; Bales et al., 2013; Hill et al., 2017), Gm08 (*Rag6*)(Zhang et al., 2017b), Gm13(*Rag2*, *Rag4*, *rag4*, and *Rag5*-proposed)(Mian et al., 2008; Zhang et al., 2009; Kim et al., 2010a; Jun et al., 2012; Bales et al., 2013; Wang et al., 2015; Hill et al., 2017), and Gm16(*Rag3*, *rag3*, *rag3b*, and *Rag3c*)(Zhang et al., 2010; Bales et al., 2013; Zhang et al., 2013; Hill et al., 2017; Zhang et al., 2017a)(reviewed by Neupane et al. (2019a). Additionally, four quantitative trait loci (QTL) have been reported on chromosomes Gm07(*qChrom.07.1*), Gm13(*qChrom.13.1*), Gm16(*qChrom.16.1*), and Gm17(*qChrom.17.1*)(Bhusal et al., 2017)(reviewed by Neupane et al. (2019a). The type of soybean aphid resistance can be antibiosis (adverse effect on insect biology) or antixenosis (feeding deterrence), and plants exhibiting tolerance (similar yield in the presence or absence of soybean aphids) have also been identified (Painter, 1951; Kogan and Ortman, 1978; Hill et al., 2004; Smith, 2005). Application of insecticides such as pyrethroids and organophosphates, has been used to manage soybean aphids to prevent aphid populations from reaching economic injury levels (Koch et al., 2016). However, since 2015, pyrethroid-resistant soybean aphids have been reported in Midwestern states (Iowa, Minnesota, North Dakota and South Dakota) (Koch et al., 2016; Koch et al., 2018), which indicates an urgent need for incorporation of host-based resistance in management strategies. Additionally, host plant resistance is an environmentally-friendly alternative strategy for the management of soybean aphids compared to application of insecticides.

A major limitation of utilizing host plant resistance is the discovery of virulent soybean aphid biotypes that successfully colonize aphid-resistant soybeans. In the United States, soybean aphid biotypes are classified based on their response to *Rag1* and/or *Rag2* genes. Biotype 1, the Illinois isolate, is unable to colonize soybean plants containing any *Rag* genes (Hill et al., 2004), and is “avirulent”; biotype 2, the Ohio isolate, is virulent on *Rag1* soybean, but not *Rag2* (Kim et al., 2008); biotype 3, the Indiana isolate, is virulent on *Rag2* but not *Rag1* soybean (Hill et al., 2010); and biotype 4, the Wisconsin isolate, is virulent on *Rag1*, *Rag2*, the *Rag1*+*Rag2* pyramid line (Alt and Ryan-Mahmutagic, 2013; Crossley and Hogg, 2015). Recent and future studies to genetically characterize soybean aphid biotypes will unravel mechanisms of aphid virulence on resistant soybean (Coates et al., 2020; Giordano et al., 2020).

The focus of this mini review is to discuss potential mechanisms of *Rag* gene action, opportunities to discover new *Rag* genes, and prospects for future research on host plant resistance to soybean aphids. In some instances, knowledge of the susceptible soybean-*Aphis glycines* interaction is used to explain phenomena related to the resistant soybean-*Aphis glycines* interaction. This review will not discuss the genomic locations of known *Rag* loci or QTLs since these aspects have been extensively reviewed by Neupane et al. (2019a).

## Potential Mechanisms of *Rag* Gene Action

At the phenotypic level, *Rag* genes reduce aphid populations on soybean plants by negatively affecting aphid biology or through feeding deterrence. Both single-Resistance (*R*) gene and multiple-*R* gene soybean genotypes significantly reduce soybean aphid populations (McCarville and O’Neal, 2012; Hesler et al., 2013; McCarville et al., 2014; Chandrasena et al., 2015; Ajayi-Oyetunde et al., 2016; Varenhorst et al., 2017; Zhang et al., 2018). Interestingly, multiple-*R* gene soybean genotypes had significantly lower aphid populations compared to those carrying a single-*R* gene (Wiarda et al., 2012; Ajayi-Oyetunde et al., 2016; Varenhorst et al., 2017; Zhang et al., 2018). This increased aphid resistance due to the presence of multiple *Rag* genes highlights the great potential of gene pyramiding as an aphid management strategy. Furthermore, using gene-pyramid varieties could extend their durability (Varenhorst et al., 2015b).

While *Rag* loci have been mapped and their approximate chromosomal location is known, none of the genes has yet been cloned. However, previous studies have identified nucleotide-binding/leucine-rich-repeat (NLR) genes, members of the most common *R* gene family Cui et al. (2015), as candidates for *Rag1* (*Glyma07g06890* and *Glyma07g06920*) (Kim et al., 2010b) and *Rag2* (*Glyma13g26000* and *Glyma13g25970*) (Kim et al., 2010a; Brechenmacher et al., 2015). In addition to candidate NLR genes, other genes have also been proposed in respective genomic regions, but all candidate genes and their mechanism of action are yet to be tested. Functional studies will be critical for the successful identification and future utilization of *Rag* genes in soybean breeding programs.

Electrical penetration graph (EPG) studies reported differences in feeding behavior of soybean aphids colonizing resistant and susceptible plants. During a 9-h period of feeding, the average time for the stylet to reach the first sieve element was shorter (~3.5 h) in the aphid-susceptible soybean genotype but longer (~7.5 h) in the aphid-resistant soybean genotype (Diaz-Montano et al., 2007; Crompton and Ode, 2010; Chandran et al., 2013). Additionally, the total duration of stylets in the sieve tube elements and phloem was longer (>1 h) in the susceptible genotype but only 2–7 min in resistant soybean genotypes and fewer aphids reached the sieve tube elements in resistant plants (Diaz-Montano et al., 2007; Chandran et al., 2013).

Insect colonization on plants triggers gene expression changes that mount defense responses consisting of morphological changes and biochemical defenses (Fernandes, 1994; Farha et al., 2010; War et al., 2012; Furstenberg-Hagg et al., 2013). Transcriptome analysis studies have been conducted for soybean genotypes carrying *Rag1*, *Rag2* (both providing antibiosis-type resistance), and *Rag5* (antixenosis). In response to aphid feeding, a rapid and strong response in resistant plants (between 4 and 48 h) was observed, while a resistance response was not observed at the transcriptome level at later time points (7 or 21 days of aphid feeding), although only *Rag1* was analyzed for these prolonged infestations (Li et al., 2008; Studham and MacIntosh, 2013; Brechenmacher et al., 2015; Lee et al., 2017; Hohenstein et al., 2019). An interesting biphasic response, with a maximum number of proteins or transcripts differentially expressed (DE) at 8 h post aphid feeding, a weak 24 h response, and another peak at 48 h was observed for *Rag2* (Brechenmacher et al., 2015). It is currently not known if *Rag1* and *Rag5* induce similar biphasic responses, as *Rag1* has not been analyzed 48 h post infestation, and *Rag5* has not been analyzed at 24 h post aphid feeding. Stated collectively, the transcriptional resistant response to soybean aphid feeding involves upregulation of transcripts involved in cell wall modification, plant defense, hormone metabolism, stress signaling, secondary metabolism, and downregulation of transcripts involved in photosynthesis and carbon metabolism (Li et al., 2008; Studham and MacIntosh, 2013; Brechenmacher et al., 2015; Lee et al., 2017). Changes in gene expression for phytohormone biosynthesis and signaling transcripts mainly jasmonic acid (JA), salicylic acid (SA), and ethylene (ET) to feeding by soybean aphids were evident, and gene expression patterns indicated cooperative action of JA and SA.

Analysis of the *Rag2* response was conducted using transcriptome and proteome analyses (Brechenmacher et al., 2015). The *Rag2* response included suppression of photosynthesis, an increase in primary and secondary cell wall metabolism, and the activation of secondary metabolism, including a large number of transcripts associated with the phenylpropanoid pathway. No clear phytohormone signature was observed, although a large number of ethylene-related transcripts were DE (both up and downregulated). Lack of correlation between DE protein and transcript abundance suggested an important role for transcriptional regulation in the *Rag2* response, an observation supported by the large number of DE transcripts related to RNA metabolism upregulated in the resistant line in response to aphid feeding (Brechenmacher et al., 2015). The *Rag5* response activated jasmonate and reactive oxygen species signaling and showed upregulation of the phenylpropanoid pathway including secondary cell wall synthesis (Lee et al., 2017). The *Rag1* response resembles a hypersensitive response and is, at least in part, mediated by salicylate signaling, and also affects cell wall, and increases the activity of the phenylpropanoid pathway (Li et al., 2008; Studham and MacIntosh, 2013).

Transcriptome comparisons between near-isogenic lines with or without the individual *Rag* genes in the absence of aphids detected DE genes (Studham and MacIntosh, 2013; Brechenmacher et al., 2015; Lee et al., 2017), leading to the suggestion that the presence of *Rag1*, *Rag2*, or *Rag5* causes constitutive expression of some defense responses (Studham and MacIntosh, 2013; Lee et al., 2017). Moreover, it is apparent that the salicylate response is primed in *Rag1* plants (Studham and MacIntosh, 2013).

A common theme among these *Rag* responses is the induction of genes related to secondary metabolite production, and it is well-documented that chemical defenses are a key plant response against aphids (Zust and Agrawal, 2016). In susceptible (non-*Rag*) soybean, long-term colonization led to upregulation of genes in the phenylpropanoid pathway, and the isoflavone daidzein has a deterrent effect on soybean aphids (Hohenstein et al., 2019). A correlation between QTL associated with soybean aphid resistance and loci associated with high isoflavone content was reported (Meng et al., 2011). Moreover, aphids feeding on *Rag1* plants induce a set of genes associated with detoxification, indicating that aphids colonizing these resistant plants are under xenobiotic stress (Bansal et al., 2014). Thus, it is possible that isoflavones or other chemical defenses are employed in the resistant response. Future studies should examine the role of chemical defenses for aphid-resistant soybean genotypes by quantifying defense-related metabolite levels such as isoflavonoids, phenolics, and others. Correlating specific metabolites and changes in aphid feeding behavior in resistant plants will advance knowledge on host plant resistance.

The effect of pyramiding *Rag* genes has also been studied at the transcriptome level (Ibore, 2017). Compared to soybean genotypes with *Rag1* or *Rag2* genes alone, the *Rag1*+*Rag2* pyramid line had a greater number of DE genes, distinctive gene sets, and activation of unique biological processes (Ibore, 2017). In the distinctive *Rag1*+*Rag2* response, there was a significant increase in defense transcripts involved in phytohormone (JA and SA) biosynthesis and signaling, secondary cell wall biogenesis, regulation of plant-type hypersensitive response, regulation of hydrogen peroxide metabolism, incompatible interaction, systemic acquired resistance, and MAPK cascade, and DE genes were mainly upregulated. A concomitant repression of all photosynthesis-related transcripts (chlorophyll biosynthesis), was observed in the *Rag1*+*Rag2* response, 6 h after aphid feeding. Differential expression of transcripts involved in secondary cell wall biogenesis can enforce stronger physical barriers to prevent further aphid colonization. The cell wall is a physical barrier that must be overcome by insects or pathogens (Bellincampi et al., 2014; Malinovsky et al., 2014), and increased cell wall thickness and lignification can prevent successful insect colonization (War et al., 2012). Reinforcement of cell walls with various macromolecules such as lignin, cellulose, and callose, occurs during insect feeding (Furstenberg-Hagg et al., 2013). Mechanisms of cell wall modification in the defense against soybean aphids are yet to be functionally characterized. However, differences in the feeding behavior of soybean aphids colonizing resistant and susceptible plants reported by EPG studies suggest that phloem-based defenses could be related to physical barriers to feeding (Diaz-Montano et al., 2007; Crompton and Ode, 2010; Zhu et al., 2011; Chandran et al., 2013).

Genes that were DE early only in the pyramid line could explain the increased aphid resistance for the *Rag1*+*Rag2* pyramid line observed at the phenotypic level. The increased aphid resistance in the *Rag1*+*Rag2* pyramid line could be caused by activation of different subsets of genetic pathways by the *Rag1* and *Rag2* genes that act synergistically to induce unique and more effective defenses against soybean aphids. While initial reports have examined effects of pyramiding *Rag* genes at the transcriptome level, additional studies are needed to fully characterize specific gene sets that contribute to increased aphid resistance, the endpoint chemical and physical responses triggered by *Rag* genes, and the functional basis of improved crop performance by *R* gene pyramiding to further improve host plant resistance. It will be important to also characterize molecular mechanisms of resistance for soybean genotypes carrying other *Rag* genes.

Another phenomenon that needs more research is induced susceptibility, in which prior colonization by virulent aphid biotypes facilitates later colonization by other aphid biotypes (Varenhorst et al., 2015a). While the mechanisms by which aphids suppress resistance are still unknown, promising induced susceptibility studies with *Rag1* soybean have been conducted by Neupane et al. (2019b), and future analyses will unravel induced susceptibility mechanisms at the transcriptome level.

A clearer understanding of potential mechanisms of *Rag* gene action will enhance development of more durable aphid-resistant soybean genotypes that can effectively control virulent aphid biotypes. When developing soybean genotypes with multiple *Rag* genes, parent genotypes must be carefully chosen to take advantage of their unique individual advantages and potential synergy in gene combinations. For instance, combining *Rag* genes that primarily elicit chemical defenses and other genes that utilize physical barriers such as cell wall modification may provide durable resistance to virulent soybean aphid biotypes.

## Opportunities to Discover New Aphid Resistance Genes

In addition to known *Rag* genes, screening studies have discovered other aphid-resistant soybean genotypes, providing opportunities to discover new *Rag* genes (Bhusal et al., 2013; Bhusal et al., 2014; Hanson et al., 2016; Conzemius et al., 2019a; Conzemius et al., 2019b; Natukunda et al., 2019). New sources of aphid resistance are particularly important because certain aphid biotypes are able to colonize resistant soybean. Identification of additional sources of aphid resistance was followed by studies that aimed to explain the genetic basis of aphid resistance and discover new *Rag* genes. Three candidate gene identification studies conducted genome-wide association studies (GWAS) (Chang and Hartman, 2017; Hanson et al., 2018; Natukunda et al., 2019). **Table 1** lists 69 aphid-resistant soybean plant introductions (PIs) included in GWAS studies, carrying resistance to biotypes 1, 2, and 3 that are prospective sources of new *Rag* genes. Additionally, across the 20 soybean chromosomes, a total of 49 significant SNPs associated with aphid resistance were reported, some of which were located on chromosomes with no reported *Rag* genes (**Table 2**).

**Table 1:** Aphid-resistant soybean genotypes (N=69) tested in genome-wide association studies (GWAS).

Soybean accession	PI is resistant to aphid biotype
***Glycine max***	
PI 437658	1^A^
PI 605765 B	1^A^
PI 157492	1^A^
PI 606394	1^A^
PI 606390 A	1^A^
PI 606398	1^A^
PI 250844	1^A^
PI 437282	1^A^
PI 592389	1^A^
PI 437353	1^A^
PI 438118	1^A^
PI 358317 B	1^A^
PI 561285 B	1^A^
PI 639534 A	1^A^
PI 639537	1^A^
PI 578388 B	1^A^
PI 507713	1^A^
PI 340034	1^A^
PI 274207	1^A^
PI 86116	1^A^
PI 248514	1^A^
PI 612759 B	2^A^
PI 171506	2^A^
PI 430491	2^A^
PI 603426 D	2^A^
PI 646911	2^A^
PI 603432 B	2^A^
PI 200595	2^A^
PI 603587 A	3^A^
PI 567250 A	3^A^
PI 603326	3^A^
PI 603339 A	3^A^
PI 153214	3^A^
PI 189946	3^A^
PI 437075	3^A^
PI 567597 C	2 and 3^A^
PI 603712	2 and 3^A^
PI 378663	1 and 3^AB^
PI 612759 C	1^AB^
PI 054854	1^B^
PI 438031	1^B^
PI 603337 A	1^B^
PI 578374	1^B^
PI 540739	1^B^
PI 603546 A	1^B^
PI 612711 B	1^B^
PI 417513 B	1^B^
PI 437950	1^B^
PI 096162	1^B^
***Glycine soja***	
PI 549046	1^A^
PI 483464 A	1^A^
PI 468397 A	1^A^
PI 468399 C	1^A^
PI 479749	1^A^
PI 479747	1^A^
PI 507786	1^A^
PI 522232	1^A^
PI 522219 A	1^A^
PI 522228	1^A^
PI 507749	1^A^
PI 507844 A	1^A^
PI 507828	1^A^
PI 507767	1^A^
PI 507838 A	1^A^
PI 507756	1^A^
PI 507741 A	1^A^
PI 507826	1^A^
PI 507840	1^A^
PI 507825	1^A^

Letters next to aphid biotypes denote reference articles that reported soybean accessions. ^A^ Hanson et al. (2018); ^B^ Natukunda et al. (2019).

**Table 2:** SNPs reported by genome-wide association studies (GWAS).

Chromosome	SNP	Minor allele	SNP position	Reference article
1	ss715578827	C	2,637,003	Hanson et al., 2018
1	ss715580619	A	55,775,590	
2	ss715583602	A	5,475,047	
4	ss715589122	C	6,142,596	
5	ss715590206	G	24,133,841	
5	ss715590836	G	33,212,449	
5	ss715590997	A	34,337,698	
6	ss715594602	C	46,884,182	
6	ss715594619	G	46,950,450	
7	ss715596585	G	1,671,208	
7	ss715596894	A	2,530,979	
7	ss715598285	G	5,062,637	
7	ss715597329	C	35,436,934	
7	ss715596142	T	11,259,155	Chang and Hartman, 2017
8	ss715599482	C	13,783,090	Hanson et al., 2018
8	ss715599561	G	14,338,011	
8	ss715600535	A	20,464,889	
8	ss715600829	C	22,052,131	
8	ss715601800	T	41,031,762	
9	ss715603059	T	1,431,512	
10	ss715606645	T	38,676,101	
10	ss715607270	T	43,371,238	
10	ss715607701	A	47,716,772	
10	ss715608208	T	51,462,329	Hanson et al., 2018 and Natukunda et al., 2019
10	ss715605620	C	1,421,982	Natukunda et al., 2019
11	ss715609271	G	25,347,421	Hanson et al., 2018
12	ss715612718	T	36,995,143	
12	ss715613201	C	5,693,819	Natukunda et al., 2019
12	ss715613209	G	5,808,606	
13	ss715614449	T	27,392,456	Hanson et al., 2018
13	ss715614803	G	29,459,954	
13	ss715614932	T	30,186,161	
13	ss715615008	C	30,654,291	
13	ss715615024	A	30,724,301	
13	ss715615352	T	32,859,112	
13	ss715615402	C	33,280,297	
13	ss715616460	G	43,544,806	
13	ss715616609	C	45,558,151	
14	ss715617401	C	10,274,971	Hanson et al., 2018
14	ss715618940	T	43,805,410	
16	ss715625258	C	6,093,779	
16	ss715624134	T	29,528,105	
17	ss715628067	T	5,888,944	
18	ss715631460	C	49,223,187	
19	ss715634601	A	228,660	Hanson et al., 2018 and Natukunda et al., 2019
19	ss715635565	T	46,220,139	Hanson et al., 2018
19	ss715635663	A	47,348,833	
19	ss715635693	G	47,552,973	
20	ss715637718	C	36,626,029	

Chromosome 13, on which the *Rag2*, *Rag4*, *rag4*, and *Rag5* genes have been reported, had the highest number of SNPs (N=9) (**Table 2**). Hanson et al. (2018) and Natukunda et al. (2019) detected the same two significant SNPs (ss715608208 and ss715605620) associated with aphid resistance (**Table 2**), confirming the usefulness of genome-wide markers for detecting candidate genes. Due to the low number of aphid-resistant soybean genotypes included in the Chang and Hartman (2017) GWAS study, only one significant SNP (ss715596142) was detected. This SNP was located on Gm07, was not close to *Rag1*, and the genomic region contained three LRR-containing genes, and one MYB transcription factor (Chang and Hartman, 2017).

Although GWAS studies have detected genomic regions associated with aphid resistance, no additional studies have validated candidate *Rag* genes for each identified soybean genotype, yet this knowledge is critical prior to utilization of new resistant soybean genotypes to develop aphid-resistant varieties. To address this knowledge gap and accelerate utilization of identified resistant soybean genotypes in plant breeding programs, future studies should identify and validate aphid-resistance genes for each resistant soybean genotype based on the location of reported significant SNPs. Wild soybeans (*Glycine soja*) are another great genetic reservoir for resistance genes (Kofsky et al., 2018), and can be utilized in plant breeding programs to develop aphid-resistant soybean varieties. For instance, PI 65549, PI 101404A, and “85-32” conferred resistance against soybean aphids (Hesler, 2013; Zhang et al., 2017a; Conzemius et al., 2019b). **Table 1** lists 20 additional wild soybean accessions that are resistant to soybean aphid biotype 1.

## Prospects

Since their discovery, soybean aphids have become a major challenge for soybean production worldwide. A clear understanding of phenotypic, transcriptional, and molecular mechanisms by which *Rag* genes confer aphid resistance is critical for development of soybean genotypes with stronger and more durable resistance. However, the mechanisms of *Rag* gene action are not fully understood, and additional studies are required to further understand the resistant soybean-*Aphis glycines* interaction. Cloning and functional validation of *Rag* genes is still the priority. Since only *Rag1*, *Rag2*, *Rag5*, and the *Rag1*+*Rag2* pyramid line have been studied at the transcriptome level, soybean genotypes carrying other known *Rag* genes, and pyramid lines with more than two genes also need to be studied. Knowledge of the mechanistic variability for the different *Rag* genes will be useful for guiding future gene pyramiding efforts, as combining different modes of killing action can lead to enhanced durability. Additionally, genes and gene networks reported by transcriptome analysis studies should be utilized to identify and validate candidate genes and genetic markers for aphid resistance traits in marker-assisted plant breeding. Soybean genotypes confirmed to be resistant to biotype 4 aphids (*Glycine max*: PI 437696 and PI 606390A; *Glycine soja*: PI 65549 and PI 101404A) (Conzemius et al., 2019a; Conzemius et al., 2019b; LaMantia et al., 2019) potentially carry new *Rag* genes and should be utilized in breeding programs to develop aphid-resistant soybean varieties. Although this mini review does not discuss the biology of the soybean aphid itself, ongoing studies to genetically distinguish the four biotypes are critical to determine distinguishing factors that make certain aphid biotypes virulent on resistant soybean. Publication of the updated version of the *Aphis glycines* genome provides a reliable reference and enhances the ability to detect biotype variability, advancing biotype-specific genetic studies (Giordano et al., 2020; Mathers, 2020). Future studies should also examine virulence of aphid biotypes on resistant soybean and utilization of additional sources of resistance to develop soybean cultivars that confer resistance to the most virulent aphid biotype 4. Association studies have laid a foundation for characterizing the genetic architecture of resistance for understudied aphid-resistant soybean genotypes, providing opportunities to use SNP markers for marker-assisted selection in breeding programs.

## Author Contributions

MIN conceptualized and prepared this manuscript. GCM intellectually contributed to and edited the manuscript.

## Funding

Iowa State University paid for publication of this mini review article through the Library Open Access Agreements with the Frontiers Journals (https://open.lib.iastate.edu/open-access/agreements).

## Conflict of Interest

The authors declare that the research was conducted in the absence of any commercial or financial relationships that could be construed as a potential conflict of interest.
